# Common and important ocular surface conditions

**Published:** 2017-02-10

**Table:** 

Condition	History and signs	Primary level management
**Infections conditions**		
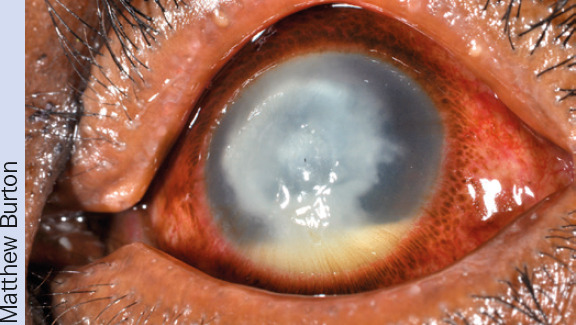 **Microbial keratitis**	**History:** Painful, red eye with reduced vision developing acutely over one or two days (bacterial) or sub-acutely over a few days (fungal). **Signs:** Corneal ulcer (epithelial defect) with underlying stromal infiltrate. The conjunctiva will be red. There may be inflammatory cells in the anterior chamber, progressing to a hypopyon in severe disease.	Hourly antibiotic eye drops and refer to a specialist.
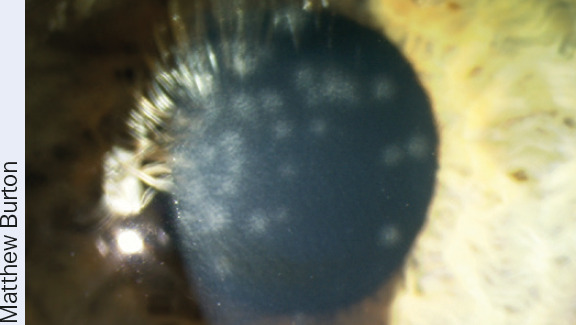 **Viral conjunctivitis**	**History:** Red, watering eyes, often bilateral. Normal or reduced vision. Mild pain. May have associated sore throat and runny nose. **Signs:** Watery discharge, conjunctival injection, tarsal conjunctival follicles, pre-auricular lymphadenopathy and eyelid oedema. The cornea may be affected with multiple superficial sub-epithelial infiltrates (grey-white spots-see image).	Avoid spread to others through good hygiene. Self-limiting.
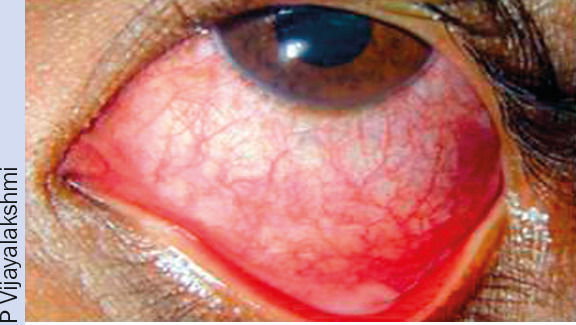 **Bacterial conjunctivitis**	**History:** Red, uncomfortable eyes with purulent discharge. There is usually redness, grittiness and burning, which may initially have been unilateral but often becomes bilateral. Lids are often stuck together in the morning with dried discharge. **Signs:** Conjunctival injection, papillary conjunctivitis, discharge.	Avoid spread to others through good hygiene. Topical antibiotics for 5–10 days.
**Allergic conjunctivitis**		
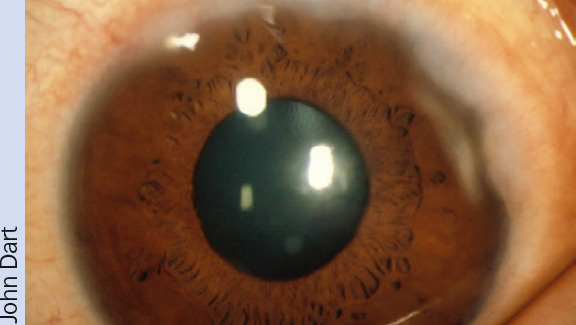 **Vernal keratoconjunctivitis (VKC)**	**History:** Allergic conjunctivitis can present at any age as itching and watering due to some known or unknown allergen. Asevere form is VKC which presents in childhood with severe itching, watering, foreign body sensation and thick mucus discharge. **Signs:** There is conjunctival injection (see image). Papillae are found in the tarsal conjunctiva, which can be large and irregular (cobblestone papillae). Tranta's spots are small white dots at the limbus. The limbus can become pigmented. The cornea can be affected with plaques and ulcération of the upper cornea.	Avoid allergens. Offer antihistamines, mast cell inhibitors, and/or topical steroids (short-term).
**Blepharitis**		
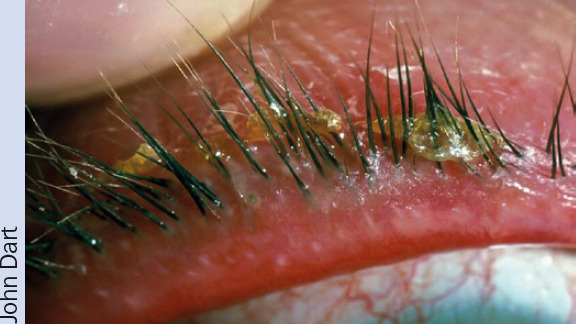 **Anterior blepharitis** 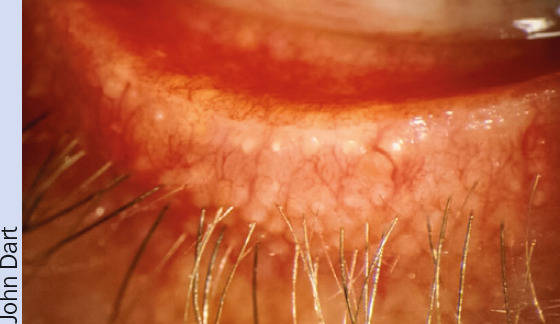 **Posterior blepharitis**	**History:** Itching, burning, uncomfortable eyes, with or without associated watering and **dry eye** symptoms (see below). There may be an associated history of recurrent meibomian cysts. **Signs:** Hard scales and crusting at the bases of lashes in anterior blepharitis. Look for capped or plugged meibomian gland orifices and hyperaemia (redness) of the posterior lid margin in posterior blepharitis.	**Anterior:** Lid cleaning to remove crusts. **Posterior:** Hot compresses and lid massage.
**Dry eye**		
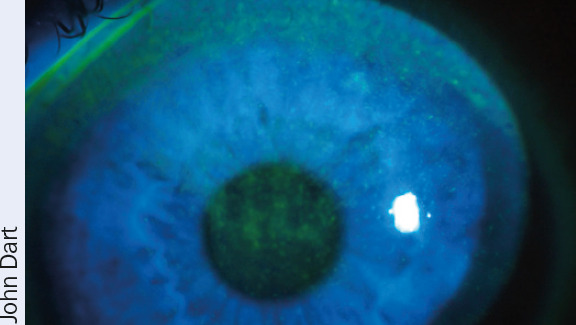 **Dry eye**	**History:** Uncomfortable, gritty eyes with a foreign body sensation. Severe cases may be photophobic and painful with reduced vision. **Signs:** The tear film is abnormal with debris on the surface and a tear break-up time of less than 10 seconds. The tear meniscus may also be thin. Punctate epithelial erosions that stain with fluorescein are the hallmark of dry eye disease.	Topical artificial tears (lubricants).
**Other inflammatory conditions**		
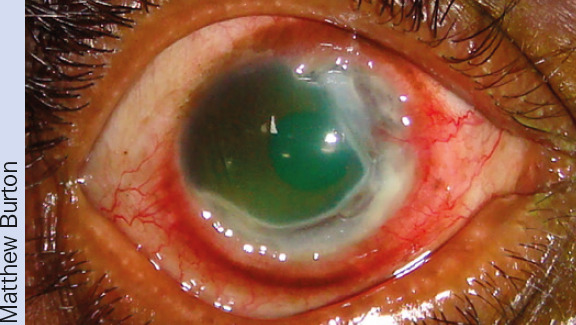 **Peripheral ulcerative keratitis (including Mooren's ulcer)**	**History:** Painful, red eye with loss of vision, developing gradually over several weeks. May have a history of systemic inflammatory disease. Mooren's ulcer is an isolated ocular problem, typically occurring in young males. **Signs:** Progressive, circumferential stromal thinning and ulceration. The limbus is inflamed in the area next to the ulceration.	Treat as for **microbial keratitis** (see above) and refer to a specialist.
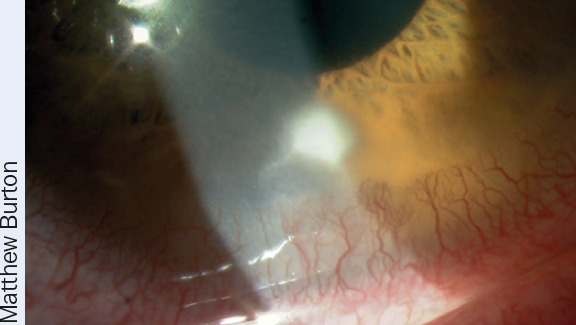 **Marginal keratitis**	**History:** Moderate pain, mild visual disturbance and redness. **Signs:** Blepharitis, subepithelial marginal infiltrates (can be multiple) with an area of clear cornea between the infiltrate and the limbus. There may be an epithelial defect, which is usually smaller than the infiltrate.	Treat initially as for **microbial keratitis.** If the diagnosis is confirmed, prescribe a low-dose topical steroid.
**Other non-inflammatory conditions**		
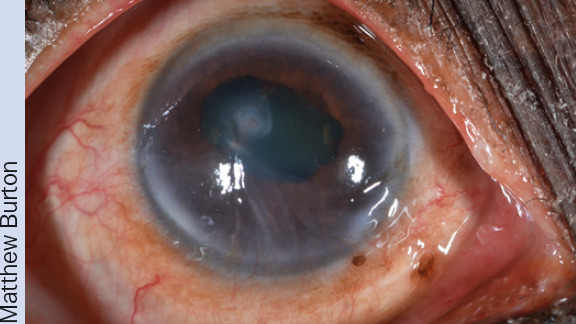 **Neurotrophic keratitis**	**History:** This should be considered in the context of systemic conditions (e.g. leprosy) or an ocular cause (e.g. herpetic keratitis or herpes zoster). The patient presents with a red eye with reduced vision. There may or may not be pain. **Signs:** Interpalpebral punctate epithelial erosions, persistent epithelial defects, stromal oedema and infiltration.	Treat the underlying cause. Protect cornea with lubricants, taping the eyelid closed at night, or lid closure.
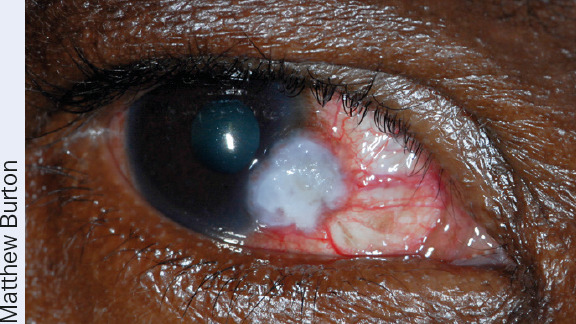 **Ocular surface squamous neoplasia**	**History:** Patients usually present with an awareness of a growing lesion on the ocular surface. This may be uncomfortable or red. There may be pain and reduced vision when large. There may be an association with HIV+ status. **Examination:** Thickened conjunctival epithelium that may extend onto the cornea with prominent ‘feeder’ vessels. There may be surface keratinisation characterised by white patches (leukoplakia), a gelatinous appearance, inflammation or pigmentation.	Refer for wide surgical excision.
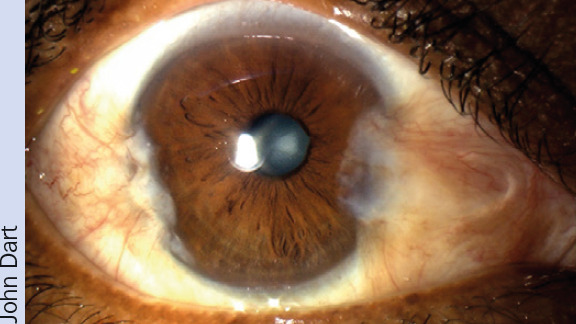 **Pterygium**	**History:** The patient may complain of a red lump, on one or both sides of the cornea, which can occasionally become more inflamed and uncomfortable. There maybe blurring of vision, depending on the extent of growth across the cornea, and induced astigmatism. **Examination:** There is a fleshy, wing-shaped growth, arising from the conjunctiva, that grows across the cornea.	Surgical excision if vision is threatened.

